# Overexpression of Long Non-coding RNA 4933425B07*Rik* Causes Urinary Malformations in Mice

**DOI:** 10.3389/fcell.2021.594640

**Published:** 2021-02-19

**Authors:** Lihong Tan, Minghui Yu, Yaxin Li, Shanshan Xue, Jing Chen, Yihui Zhai, Xiaoyan Fang, Jialu Liu, Jiaojiao Liu, Xiaohui Wu, Hong Xu, Qian Shen

**Affiliations:** ^1^Department of Nephrology, Shanghai Kidney Development and Pediatric Kidney Disease Research Center, Children’s Hospital of Fudan University, Shanghai, China; ^2^State Key Laboratory of Genetic Engineering and National Center for International Research of Development and Disease, Institute of Developmental Biology and Molecular Medicine, Collaborative Innovation Center of Genetics and Development, School of Life Sciences, Fudan University, Shanghai, China

**Keywords:** long non-coding RNA, *4933425B07Rik*, congenital anomalies of the kidney and urinary tract, kidney development, *Bmp4*

## Abstract

Congenital anomalies of the kidney and urinary tract (CAKUT) is a common birth defect and is the leading cause of end-stage renal disease in children. The etiology of CAKUT is complex and includes mainly genetic and environmental factors. However, these factors cannot fully explain the etiological mechanism of CAKUT. Recently, participation of long non-coding RNAs (lncRNAs) in the development of the circulatory and nervous systems was demonstrated; however, the role of lncRNAs in the development of the kidney and urinary tract system is unclear. In this study, we used the *piggyBac* (PB) transposon-based mutagenesis to construct a mouse with lncRNA *4933425B07Rik* (*Rik*) PB insertion (*Rik*^PB/PB^) and detected overexpression of *Rik* and a variety of developmental abnormalities in the urinary system after PB insertion, mainly including renal hypo/dysplasia. The number of ureteric bud (UB) branches in the *Rik*^PB/PB^ embryonic kidney was significantly decreased in embryonic kidney culture. Only bone morphogenetic protein 4 (*Bmp4*), a key molecule regulating UB branching, is significantly downregulated in *Rik*^PB/PB^ embryonic kidney, while the expression levels of other molecules involved in the regulation of UB branching were not significantly different according to the RNA-sequencing (RNA-seq) data, and the results were verified by quantitative real-time polymerase chain reaction (RT-PCR) and immunofluorescence assays. Besides, the expression of pSmad1/5/8, a downstream molecule of BMP4 signaling, decreased by immunofluorescence. These findings suggest that abnormal expression of *Rik* may cause a reduction in the UB branches by reducing the expression levels of the UB branching-related molecule *Bmp4*, thus leading to the development of CAKUT.

## Introduction

Congenital anomalies of the kidney and urinary tract (CAKUT) are a group of developmental diseases with abnormal anatomical structure of the urinary system. The phenotype of CAKUT is broadly defined to include abnormalities of the kidneys, ureters, bladder, and urethra. CAKUT is a common developmental birth defect in children. On average, the prevalence of CAKUT varies from 3 to 6 per 1,000 newborns (Nicolaou et al., 2015), accounting for 20–30% of congenital birth defects (Murugapoopathy and Gupta, 2020). CAKUT is the leading cause of chronic kidney disease (CKD) in children, accounting for 40–50% of children receiving renal replacement therapy (Chesnaye et al., 2014), which is a heavy burden on the families of the children and on society.

The development of the mammalian urinary system includes three phases: pronephros, mesonephros, and metanephros. Metanephros eventually develops into a mature kidney. Metanephros development depends on the interaction between two types of cells: metanephric mesenchyme (MM) cells and ureteric bud (UB) cells. Metanephros development starts at embryonic day (E) 10.5. A signal from MM induces UB outgrowth from the nephric duct, and the UB then penetrates the MM. On E11.5, the UB forms the first T-shaped structure. After iterative branching, the UB eventually forms a renal collecting system; at the same time, a signal from the UB induces epithelial differentiation of MM cells with subsequent gradual nephron formation (Blake and Rosenblum, 2014; Short and Smyth, 2016). At this stage, abnormal UB outgrowth and branching leads to CAKUT. Insufficient UB outgrowth causes renal dysplasia, excessive UB outgrowth leads to a duplex renal collecting system, and reduced UB branching may result in renal hypoplasia or renal dysplasia (Kozlov and Schedl, 2020).

The etiological mechanism of CAKUT has not been fully elucidated. Currently, the classical etiological factors of CAKUT are considered to be genetic and environmental. Genetic factors included single gene mutation, repeat copy number variation, and abnormal chromosome structure, which account for 10, 15, and 20% of cases, respectively. However, etiological factors cannot be clearly defined in approximately half of the patients (Rosenblum et al., 2017). Thus, additional potential etiological factors of CAKUT have to be studied, including non-coding RNAs, histone modifications, and other epigenetic factors. Long non-coding RNAs (lncRNAs) may be one of these factors. lncRNAs are a class of non-coding RNAs with more than 200 nucleotides that lack an intact open reading frame and cannot encode proteins. In recent years, numerous studies have demonstrated that lncRNAs can regulate the developmental physiology of various tissues and organs, such as the circulatory and nervous systems. For instance, lateral mesoderm-specific lncRNA *Fendrr* anchors polycomb repressive complex 2 (*PRC2*), increasing *PRC2* occupancy and the levels of histone H3 Lys27 tri-methylation (H3K27me3) in heart development (Grote et al., 2013). Loss of the lncRNA *lncrps25* reduces locomotion behavior by regulating motor neuron progenitor development and expression of oligodendrocyte transcription factor (Olig2) (Gao et al., 2020). Expression of the MM-specific lncRNA *GM29418* can regulate SIX homeobox 2 (*Six2*) in mouse MM cells *in vitro*, and *Six2* has been demonstrated to regulate the development of the metanephros (Nishikawa et al., 2019). Expression of the lncRNA *Hoxb3os* (homeobox B3 and homeobox B2, opposite strand) is significantly decreased in the renal tissues of patients with autosomal dominant polycystic kidney disease (ADPKD) and in a mouse model of ADPKD (Aboudehen et al., 2018). Thus, we hypothesized that the presence of abnormal lncRNAs may lead to abnormal development of the urinary system and may induce CAKUT in a mouse model.

The intergenic lncRNA *Rik* is located at 14qC1: 46,602,806-46,637,423. A review of the literature found that abnormal expression of the lncRNA *Rubie* (14qC1: 46,568,263-46,587,983) located adjacent to *Rik* can cause vestibular malformations in the inner ear of mice. Thus, we hypothesized that *Rik* may participate in embryonic development including the urinary system development in mice. Therefore, in this study, we used the *piggyBac* (PB) transposon-based insertional mutagenesis to construct the lncRNA *4933425B07Rik* (*Rik*) PB to insert in mice and generate a mutant mouse model (*Rik*^PB/PB^) to investigate the phenotype and the possible mechanism.

## Experimental Materials and Methods

### Mice

*Rik*^PB/+^ mice were obtained by using PB transposon-based insertional mutagenesis targeted to intron 3 of the *Rik* gene of FVB/NJ mice (Ding et al., 2005). *Rik*^PB/+^ mice were inbred to obtain *Rik*^PB/PB^ and *Rik*^+/+^ mice. Hoxb7/myr-Venus is a previously successfully bred mouse strain that specifically expresses a fluorescent protein in the UB epithelium (Yu et al., 2020). *Rik*^PB/PB^ mice were mated with Hoxb7/myr-Venus mice to obtain *Rik* PB insertion mice specifically expressing fluorescent protein in the UB epithelium (*Rik^PB/+^*; *Hoxb7*/myr-Venus abbreviated as *Rik^PB/+^*; *Hoxb7*). *Rik*^PB/+^; *Hoxb7* were inbreed to obtain offspring namely Rik^PB/PB^; Hoxb7/myr-Venus (Rik^PB/PB^; Hoxb7), Rik^PB/+^; Hoxb7, and control mice Rik^+/+^; Hoxb7/myr-Venus (Rik^+/+^; Hoxb7). We observed the mice urinary system phenotypes by selected the same nest of different genotypes. All mice used in the experiments were specific pathogen free (SPF) and were maintained at 18–22°C, 50–60% humidity, and 12 h day/night cycle. Animals were maintained and managed according to the animal welfare and usage management regulations of the School of Life Sciences of Fudan University [Protocol Approval No. SYXK (hu) 2020-0011].

### RNA Extraction and Quantitative Real-Time PCR

The TRIzol (Life Technologies, United States) method was used to extract RNA from E12.5 whole embryos, from E14.5, E16.5, E18.5, and postnatal day (P) 0.5 kidneys, and from E14.5 heart, liver, brain, lung, ureter, and bladder. RNeasy mini kit (QIAGEN, Germany) was used to extract RNA from E12.5 embryonic kidney. Genomic DNA removal, reverse transcription, and quantitative real-time polymerase chain reaction (RT-PCR) were performed according to manufacturer instructions. Genomic DNA was removed using a gDNA eraser kit (Takara, Japan), and reverse transcription was performed using a reverse transcription kit (Takara, Japan). RT-PCR was performed using the AceQ qPCR SYBR Green Master Mix (Vazyme, China) and a real-time qPCR system (Agilent Mx3000P, United States). RT-PCR primers for *Rik*, bone morphogenetic protein 4 (*Bmp4*), Wnt9b, forkhead box C1 (*Foxc1*), *Foxc2*, GATA-binding protein 3 (*Gata3*), cyclin dependent kinase inhibitor 3 (*Cdkn3*), glia maturation factor beta (*Gmfb*), sterile alpha motif domain containing 4 (*Samd4*), cell growth regulator with ring finger domain 1 (*Cgrrf1*), cornichon family AMPA receptor auxiliary protein 1 (*Cnih1*), and glyceraldehyde 3-phosphate dehydrogenase (*Gapdh*) are listed as follows. For *Rik*, 5′-TTGGGTGTGACTGGAGGAAA-3′ (forward) and 5′-CAAA CAGGATAAAGATGGGAGGT-3′ (reverse); for *Bmp4*, 5′-GCA AGTTTGTTCAAGATTGGCTCC-3′ (forward) and 5′-CCAT CAGCATTCGGTTACCAGG-3′ (reverse); for *Wnt9b*, 5′-TTC CCCGACACTCAGCAAAG-3′ (forward) and 5′-TTCCCCTT CTCAGTCTGTTCTCCG-3′(reverse); for *Foxc1*, 5′-GCCAAA TGGAATGGAACCCC-3′ (forward) and 5′-CGCTGGTGTGA GGAATCTTCTC-3′ (reverse); for *Foxc2*, 5′-CTTCTACCGCG AGAACAAGC-3′ (forward) and 5′-GACTTTCTTCTCGGCCT CCT-3′ (reverse); for *Gata3*, 5′-CCAGGCAAGATGAGAAA GAGTG-3′ (forward) and 5′-ATAGGGCGGATAGGTGGTA ATG-3′ (reverse); for *Cdkn3*, 5′-GAGCACATCTAGGGT CTCCA-3′ (forward) and 5′-ACACCGAATTCATGACTCA AGC-3′ (reverse); for *Gmfb*, 5′-CCAAGAGATCGCACGGC AAC-3′ (forward) and 5′-AGGCGTTCATCCTTGTCAATCT-3′ (reverse); for *Cgrrf1*, 5′-CTTCCCTAGCAACAGGCATGG-3′ (forward) and 5′-TCTTCTAACGCTTGCGGGG-3′ (reverse); for *Cnih1*, 5′-TCGCGGCCTTCTGCTATATG-3′ (forward) and 5′-GGAAGGACAAGAGGGTTCAGG-3′ (reverse); and for *Gapdh*, 5′-TGTTCCTACCCCCAATGTGTCC-3′ (forward) and 5′-GGAGTTGCTGTTGAAGTCGCAG-3′ (reverse). *Gapdh* was used as an internal control for relative quantification. The relative gene expression levels were calculated using the 2^–Δ^ ^Δ^ ^CT^ method. Each group contained at least three samples, and each sample was assayed in triplicate.

### Nuclear and Cytoplasmic Extraction

Mouse renal proximal tubular epithelial cells (mPTCs), an immortalized cell line, were acquired from ATCC and cultured in DMEM-F12 medium supplemented with 10% fetal bovine serum (FBS) (Yang et al., 2018). After collection of mPTCs, the cytoplasm and nuclei were isolated using the NE-PER Nuclear and Cytoplasmic Extraction kit (Thermo Fisher Scientific, United States). Cytoplasmic and nuclear RNA was extracted using the RNeasy kit (Qiagen, Germany). *Gapdh* mRNA, *U1* spliceosomal RNA and metastasis associated lung adenocarcinoma transcript 1 (*MALAT1*) RNA were used as controls.

### Gross Phenotypic Analysis

Phenotypes of newborn *Rik*^PB/PB^, *Rik*^PB/+^, and *Rik*^+/+^ mice were assessed. The animals were anesthetized with CO_2_ and the abdomen was dissected to expose the urinary system. The location, morphology, and number of kidneys, ureters, and bladders of mice in each group were observed under a stereoscope. The images were recorded under a microscope (Leica, Germany).

### Hematoxylin-Eosin Staining

The kidneys of the mice were removed and fixed in 4% paraformaldehyde. After ethanol gradient dehydration, the tissues were embedded in paraffin and cut into 4 μm sections. Hematoxylin-eosin (H&E) staining was performed according to the standard protocols described in the literature (Xu et al., 1999).

### Embryonic Kidney Culture

E11.5 embryonic kidneys from *Rik*^PB/PB^; Hoxb7 and *Rik*^+/+^; Hoxb7 mice were isolated in phosphate-buffered saline (PBS) and placed on a 0.4 μm polyester membrane (Corning, United States) at 37°C and 5% CO_2_. Embryonic kidneys were cultured in DMEM-F12 (Gibco, United States) containing 10% FBS (Gibco, United States) and 1% penicillin/streptomycin (Gibco, United States) for 24, 48, and 72 h and then observed under a fluorescence microscope (Leica, Germany) to detect UB branches and count the UB tips (Kim et al., 2014).

### RNA-Sequencing

The RNeasy Mini kit (QIAGEN, Germany) was used to extract the total RNA of E12.5 embryonic kidneys from the *Rik*^PB/PB^; Hoxb7 and *Rik*^+/+^; Hoxb7 mice. The sequencing data was filtered with SOAPnuke (v1.5.2) (Li et al., 2008) by (1) removing reads containing sequencing adapter; (2) removing reads whose low-quality base ratio (base quality less than or equal to 5) is more than 20%; (3) removing reads whose unknown base (“N” base) ratio is more than 5%, afterward clean reads were obtained and stored in FASTQ format. The clean reads were mapped to the reference genome using HISAT2 (v2.0.4) (Kim et al., 2015), Bowtie2 (v2.2.5) (Li and Dewey, 2011) was applied to align the clean reads to the reference coding gene set, then expression level of gene was calculated by RSEM (v1.2.12) (Raivo, 2019). Using Fragments Per Kilobase of exon model per Million mapped fragments (FPKM) algorithm for the expression of standardized, which counts by total exon fragments/[mapped reads (millions) × exon length (kb)], reflects the gene expression level. The heatmap was drawn by pheatmap (v1.0.8) (Love et al., 2014) according to the gene expression in different samples. Essentially, differentially expressed genes (DEGs) analysis was performed using the DESeq2 (v1.4.5) (Abdi, 2007) with fold change ≥2 and false discovery rate (FDR) ≤ 0.001. To take insight to the change of phenotype, GO^1^ enrichment analysis of annotated different expressed gene was performed by Phyper^2^ based on Hypergeometric test. The significant levels of terms and pathways were corrected by *Q*-value (=FDR) with a rigorous threshold (*Q*-value ≤ 0.05) by Bonferroni.

### Immunofluorescence

Fresh kidney tissue was fixed with 4% paraformaldehyde overnight, dehydrated in 30% sucrose for 48 h, embedded in optimal cutting temperature compound (OCT compound), and stored at −80°C. The sections were frozen and sliced at a thickness of 15 μm. The sections were washed with PBS, permeabilized with 0.3% Triton X-100 for 15 min, and blocked with 5% donkey serum for 1 h at room temperature. The tissue slices were incubated with a primary antibody at 4°C overnight, washed several times with PBS, incubated with a secondary antibody at room temperature for 1 h, washed several times with PBS, and treated with an autofluorescence quencher. Slices were observed and imaged using a fluorescence confocal microscope (Olympus FV3000, Japan). The following antibodies were used: primary antibodies: anti-BMP4 (Abcam, ab39973, 1:200), anti-Smad1/Smad5/Smad8 (Affinity, AF-0614, 1:100), and anti-pSmad1/pSmad5/pSmad8 (Cell Signaling Technology, CST13820, 1:200); secondary antibodies: Alexa 647-conjugated anti-rabbit (Jackson ImmunoResearch, 1:400).

### Statistical Analysis

All data were processed using the SPSS 24.0 statistical software. The data are presented as the mean ± standard deviation. The count data are presented as rates. Differences between the groups were analyzed using the Chi-squared test or unpaired *t*-test. *P* < 0.05 was defined as a statistically significant difference.

## Results

### *Rik* Overexpression in *Rik*^PB/PB^ Mice

**FIGURE 1:**
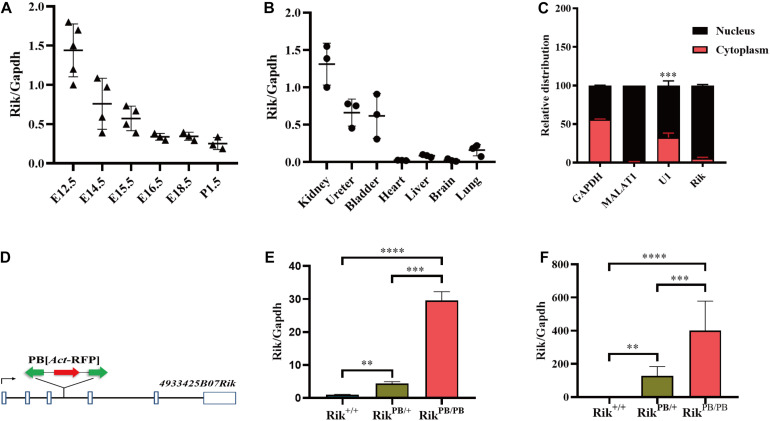
*Rik* overexpression in PB mice. **(A)** *Rik* expression during various kidney development stages in *Rik*^+/+^ mice. **(B)** *Rik* expression distribution in various tissues of *Rik*^+/+^ mice at E14.5. **(C)** Subcellular localization of *Rik*. **(D)** Genomic structure after PB insertion. PB (*Act*-RFP) was inserted into intron 3 of the *Rik* gene (solid line). Boxes, exons; arrows, transcription direction; green arrows, PB transposon; red arrows, RFP expression elements. **(E)** After PB insertion, the expression level of *Rik* was increased in E12.5 embryos, *n* = 3. **(F)** The expression level of *Rik* in E12.5 embryonic kidney was increased after PB insertion, *n* = 5. **P < 0.01; ***P < 0.001; ****P < 0.0001.

We used the phylogenetic codon substitution frequency software (PhyloCSF) to identify an open reading frame and ORF Finder to assess the protein-coding potential of *Rik*; the results demonstrated that *Rik* is a lncRNA because of its low protein-coding potential (Supplementary Figure 1). Non-coding RNAs are able to accurately regulate the development of the metanephros in a specific spatiotemporal pattern due to unique tissue and embryonic expression characteristics. We detected the spatiotemporal expression distribution of *Rik* in wild-type mice by RT-PCR and found that the expression level was high during E12.5-E15.5; expression was highest on E12.5 and then gradually decreased (Figure 1A). At E14.5, high levels of *Rik* were expressed in the mouse urinary system (kidney, ureter, and bladder), while the expression levels in the brain, heart, lung, and liver were low (Figure 1B). The subcellular localization of *Rik* in mPTC was detected using the nuclear and cytoplasmic protein extraction assay; the data indicate that *Rik* is almost exclusively present in the nucleus (Figure 1C).

*Rik*^PB/PB^ mice were constructed using PB transposon-based insertional mutagenesis. PB was inserted into intron 3 of the mouse *Rik* gene (Figure 1D), resulting in *Rik* overexpression in the *Rik*^PB/PB^ mice. RT-PCR of whole embryos and embryonic kidneys of E12.5 *Rik*^PB/PB^, *Rik*^PB/+^, and *Rik*^+/+^ mice detected an increase in *Rik* expression levels in *Rik*^PB/PB^ and *Rik*^PB/+^ mice compared to that in *Rik*^+/+^ mice (Figures 1E,F).

### *Rik*^PB/PB^ Newborn Mice Develop Urinary Malformations

**FIGURE 2:**
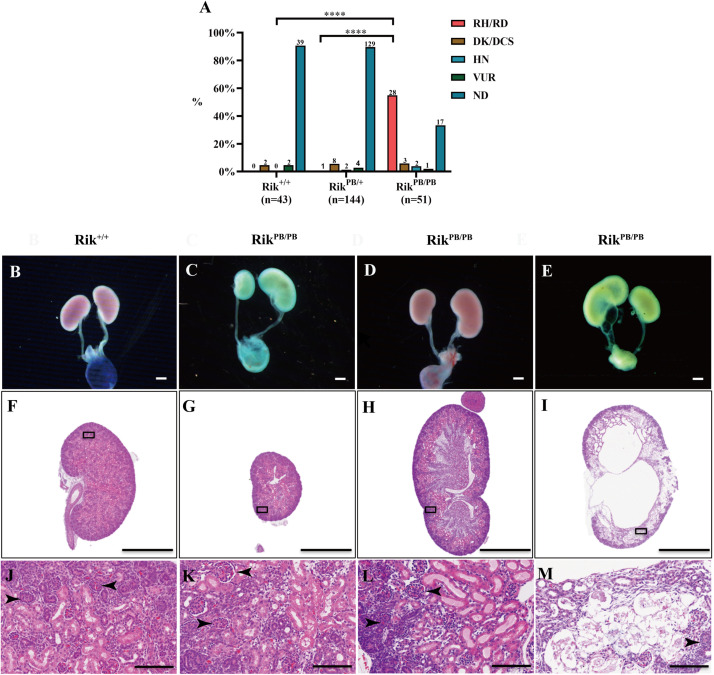
Various urinary malformations observed in *Rik*^PB/PB^ newborn mice predominantly manifested as renal hypo/dysplasia. **(A)** The percentage of urinary malformations in *Rik*^PB/PB^, *Rik*^PB/+^, and *Rik*^+/+^ newborn mice. The number n represents the number of cases. **(B–E)** Overview images of the urinary system of newborn mice. **(F–M)** H&E staining of the kidneys of newborn mice. **(J–M)** Local magnified images. **(B,F,J)** Normal kidney of *Rik*^+/+^ mice. **(C,G,K)** Renal dysplasia/hypoplasia in *Rik*^PB/PB^ mice. **(D,H,I)** Duplex kidney in *Rik*^PB/PB^ mice. **(E,I,M)** Hydronephrosis in *Rik*^PB/PB^ mice. RH/RD, renal hypo/dysplasia; DK/DCS, duplex kidneys/duplex collecting systems; HN, hydronephrosis; VUR, vesicoureteral reflux; ND, normal kidney. Arrowhead, nephron. ****P < 0.0001. Scar bars, 10 mm in **(B–E)**; 1 mm in **(F–I)**; 100 μm in **(J–M)**.

Gross phenotypes of the urinary system of *Rik*^PB/PB^, *Rik*^PB/+^, and *Rik*^+/+^ newborn mice were examined, and the results indicate a significantly higher incidence of CAKUT in *Rik*^PB/PB^ newborn mice than in *Rik*^PB/+^ mice and *Rik*^+/+^ mice (66.7%, 34/51 vs 10.4%, 15/144, *P* < 0.001; 66.7%, 34/51 vs 9.3% (4/43), *P* < 0.001, respectively, Figure 2A). The CAKUT phenotype (Figures 2B–M) of the *Rik*^PB/PB^ mice included renal hypo/dysplasia (82.4%, 28/34, Figures 2C,G,K), duplex renal/collecting system (8.8%, 3/34, Figures 2D,H,L), hydronephrosis (5.9%, 2/34, Figures 2E,I,M), and vesicoureteral reflux (VUR) (2.9%, 1/34, Figure not shown) with renal hypo/dysplasia being the most common phenotype. The renal pathology of newborn mice in the *Rik*^PB/PB^ group was examined by H&E staining, which indicated that the structure and morphology of the glomerulus and renal tubules in animals with renal hypo/dysplasia and duplex kidneys were normal (Figures 2K,L). The numbers of glomeruli and tubules were reduced in the renal tissues in animals with hydronephrosis (Figure 2M). Gross anatomy and histopathology of the brain, heart, lung, and liver of *Rik*^PB/PB^ newborn mice were observed, and no significant abnormalities were detected (Supplementary Figure 2).

### UB Branches in the Early Stages of Metanephric Development of *Rik*^PB/PB^ Mice

**FIGURE 3:**
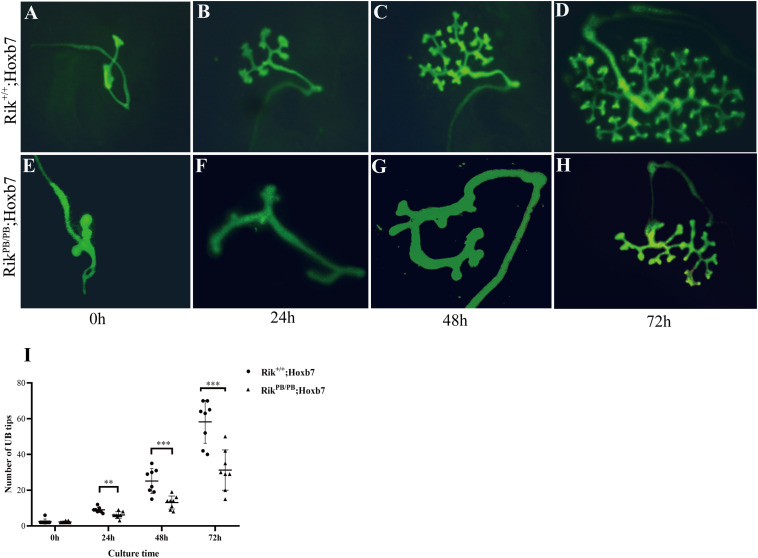
Decrease in UB branches in the embryonic kidney of *Rik*^PB/PB^. **(A,E)** Embryonic kidney at E11.5. **(B,F)** Embryonic kidney after 24 h culture. **(C,G)** Embryonic kidney after 48 h culture. **(D,H)** Embryonic kidney after 72 h culture. **(I)** The UB tips count of UB branches in wild-type and homozygous embryonic kidneys, *n* = 8. **P < 0.01; ***P < 0.001.

The effect of *Rik* on UB branching in the early stages of metanephric development was observed. E11.5 embryonic kidneys of *Rik*^PB/PB^ and *Rik*^+/+^ mice were extracted for *in vitro* culture. The number of UB tips in these two groups was counted at 24, 48, and 72 h. The number of UB branches at these three time points was significantly lower in the *Rik*^PB/PB^ group than in the *Rik*^+/+^ group (9.0 ± 1.5 vs 6.5 ± 1.8, 25.1 ± 7.0 vs 12.5 ± 3.3, and 57.9 ± 10.8 vs 31.3 ± 11.2, respectively, Figures 3A–I).

### *Rik* Overexpression Induces Abnormal Expression of Metanephric Development-Related Genes in Mice

**FIGURE 4:**
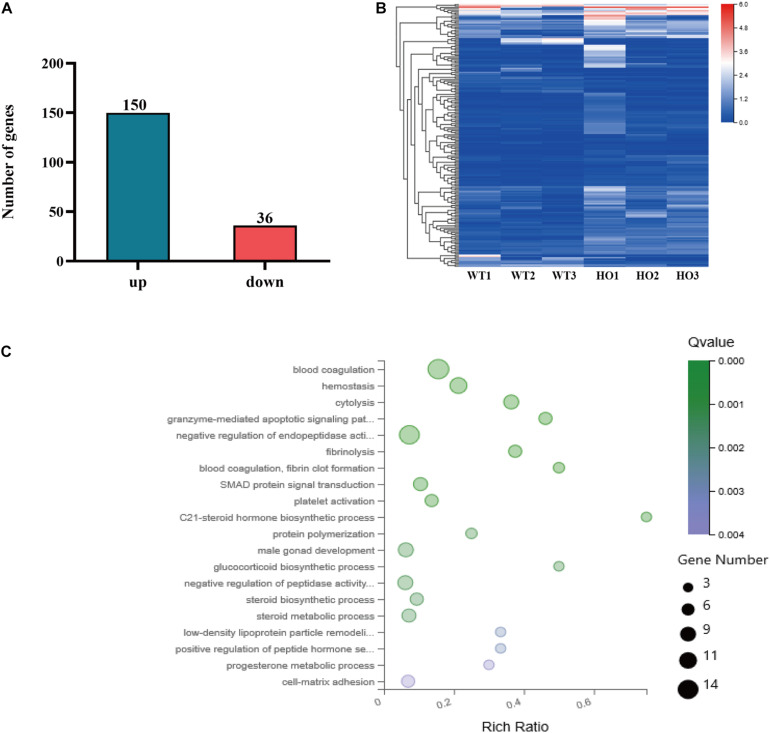
RNA-seq analysis. **(A)** Number of DEGs identified by RNA-seq. **(B)** Heat map of DEGs identified by RNA-seq. WT, wild-type namely *Rik*^+/+^; Hoxb7 mice; HO, homozygous namely *Rik*^PB/PB^; Hoxb7 mice. **(C)** Bubble plots of the gene ontology (GO) enrichment of DEGs in the E12.5 embryonic kidney [*Q*-value means false discovery rate (FDR) or adjusted *P*-value. Rich ratio is the ratio of the signaling pathway of enrichment on the number of differentially expressed genes and signaling pathways of all the genes].

To clarify the effect of *Rik* overexpression on the expression of genes related to metanephric development in mice, we extracted RNA from the kidneys of E12.5 *Rik*^+/+^ and *Rik*^PB/PB^ mice to perform RNA-sequencing (RNA-seq). The results showed that the number of DEGs in the wild-type vs homozygous mutant mice was 186, including 150 upregulated genes and 36 downregulated genes (Figures 4A,B). These 186 DEGs were classified and enriched, including the SMAD protein signal transduction pathway [*Bmp4*, transferrin (*Trf*), growth differentiation factor 2 (*Gdf2*), *Gdf10*, *Gata4*, hepatocyte nuclear factor 4 alpha (*Hnf4a*), and alpha-fetoprotein (*Afp*)], Figure 4C, which is related to the development of the urinary system. Subsequent analysis of the molecules involved in the regulation of the UB branching revealed differential expression: the upregulated genes (fold increase) included *Foxc1* (1.20), *Wnt9b* (1.24), *Foxc2* (1.18), and *Gata3* (1.18), and the downregulated genes (fold decrease) included *Bmp4* (0.48) (Supplementary Table 1).

**FIGURE 5:**
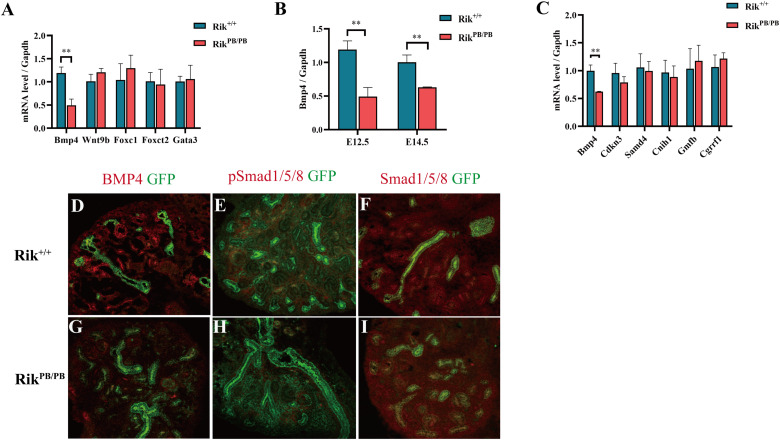
*Rik* overexpression-induced abnormal expression of genes related to mouse metanephric development. **(A)** Expression changes of the key genes in metanephric development at E12.5, *n* = 3. **(B)** Changes in *Bmp4* expression at various embryonic ages, *n* = 4. **(C)** Expression of the genes adjacent to the *Rik* gene locus. **(D–I)** Immunofluorescence was used to detect the expression of BMP4, Smad1/Smad5/Smad8 and pSmad1/pSmad5/pSmad8 in the kidneys of *Rik*^+/+^ and *Rik*^PB/PB^ mice at E14.5. *n* = 3, original magnification ×20 **(D–I)**.

Then, RT-PCR was performed to validate the DEGs regulating UB branching. The expression of *Bmp4* in the E12.5 *Rik*^PB/PB^ group was significantly decreased (Figure 5A), and the expression levels of other genes were not significantly different. RT-PCR detection revealed significantly decreased expression of *Bmp4* in the E14.5 *Rik*^PB/PB^ group (Figure 5B).

Considering that *Rik* is mainly distributed in the nucleus, we identified the genes located adjacent to *Rik* in the same region of chromosome 14, including *Bmp4*, *Cdkn3*, *Samd4*, *Cnih1*, *Gmfb*, and *Cgrrf1*. The changes in the expression levels of these genes in the kidney of E14.5 *Rik*^PB/PB^ were analyzed using RT-PCR; the results indicated that *Bmp4* was significantly downregulated (Figure 5C). Immunofluorescence analysis of *Bmp4* and its downstream targets Smad1/Smad5/Smad8 and pSmad1/pSmad5/pSmad8 indicated that the expression levels of Smad1/Smad5/Smad8 were not significantly changed in E14.5 *Rik*^PB/PB^ homozygous kidney, while the expression levels of BMP4 and pSmad1/pSmad5/pSmad8 were decreased (Figures 5D–I).

## Discussion

In this study, we successfully established *Rik*^PB/PB^ mice using PB transposon-based insertional mutagenesis and found that *Rik* is overexpressed after PB insertion. *Rik*^PB/PB^ mice had multiple developmental malformations of the urinary system predominantly manifested as renal hypo/dysplasia. Additionally, the PB insertion induced a decrease in the number of UB branches by influencing UB branching at the early stage of metanephric development. Analysis of the expression profiles of the molecules related to early UB branching in *Rik*^PB/PB^ mice revealed that the expression levels of *Bmp4*, a key gene regulating ureteral bud branching, were decreased in the *Rik*^PB/PB^ embryonic kidney. Therefore, we suggest that abnormal *Rik* expression may lead to the occurrence of CAKUT in mice and that the lesions may be mediated by altered expression of the key factor *Bmp4* during the early UB branching stages.

Previous studies have shown that lncRNAs are related to the development of the mouse urinary system. Nishikawa et al. identified a lncRNA, *Gm29418*, that was specifically expressed in MM cells in mice. *Gm29418* has an enhancer-like function on *Six2 in vitro*, which is the key regulatory gene in MM cells (Nishikawa et al., 2019). In another study, *Hoxb3os*, a kidney-specific, evolutionarily conserved lncRNA, was shown to be downregulated in the cyst epithelium in an ADPKD adult mouse model. The data indicate that *Hoxb3os* can regulate the signaling of mammalian target of rapamycin complex 1 (mTORC1) and mitochondrial respiration *in vitro* (Aboudehen et al., 2018). However, causal associations between abnormal lncRNA and CAKUT occurrence have not been confirmed in animal models. In this study, *Rik*-overexpressing mice were established and were shown to develop various phenotypes of urinary malformations predominantly manifested as renal hypo/dysplasia. The biological functions of lncRNAs are closely related to their tissue specificity and temporal and spatial specificity. This study demonstrates that *Rik* is specifically expressed in the mouse kidneys and that the expression level was increased in the early stages of metanephric development. These findings suggest that *Rik* regulates the development of the urinary system in mice, and abnormal *Rik* expression may lead to CAKUT.

The normal development of the mouse urinary system is closely associated with UB outgrowth and branching. UB outgrowth begins on E10.5, and the early branching stage occurs on E11.5–E15.5 (Short and Smyth, 2016). In this process, abnormal or ectopic UB outgrowth or abnormal position of the outgrowth may lead to renal agenesis, duplex kidney, hydronephrosis, or VUR; reduction in UB branches or early termination of the branching may result in renal hypo/dysplasia (Schedl, 2007). In this study, *Rik*^PB/PB^ mice predominantly manifested renal hypo/dysplasia. Dynamic observation of the UB branching process in *in vitro* culture showed that the number of UB branches in the *Rik*^PB/PB^ embryonic kidney was significantly reduced. Iterative UB branching is regulated by a complex molecular network, and abnormalities of certain key molecules may determine the lesions of the UB branches. Previous studies have shown that the abnormal expression of genes [Bmp4 (Chang et al., 2008), Foxc1 (Kume et al., 2000), Wnt9b (Carroll et al., 2005), Gata3 (Grote et al., 2008), Six2 (Self et al., 2006), Ret (Allison, 2012), Fgf7 (Qiao et al., 1999), Gremlin1 (Michos et al., 2004) etc.] can lead to a reduction in UB branches. This study used RNA-seq for the preliminary screening and RT-PCR and immunofluorescence to verify the changes in various molecules and found that only *Bmp4* is significantly downregulated, while the expression levels of other molecules involved in the regulation of UB branching were not significantly different from *Rik^+/+^* embryonic kidney. BMP4 belongs to the transforming growth factor beta (TGF-β) superfamily and participates in urinary system development by regulating cell proliferation, differentiation, and apoptosis (Hogan, 1996; von Bubnoff and Cho, 2001). Renal hypo/dysplasia has been observed in patients with BMP4 mutations (Bakrania et al., 2008; Weber et al., 2008; Davis et al., 2014). Moreover, the CAKUT phenotype predominantly manifested as renal hypo/dysplasia has been observed in *Bmp4*^±^ mice (Miyazaki et al., 2003), similar to the CAKUT phenotype of *Rik*^PB/PB^ mice in the present study. Extracellular BMP4 can interact with BMP receptor type I or II (BMPR1/BMPR2), transmembrane serine/threonine kinase receptors, to form a complex, which triggers the signal transduction cascade of intracellular Smad1/Smad5/Smad8 protein phosphorylation, thereby regulating cellular processes (Bakrania et al., 2008). Studies of the mechanisms of renal hypo/dysplasia suggest that renal hypo/dysplasia may be associated with abnormal phosphorylation of the downstream molecules of BMP4 signaling (van der Ven et al., 2018). The present study also detected a significant reduction in the expression of pSmad1/pSmad5/pSmad8, which are located downstream of *Bmp4*, in *Rik*^PB/PB^ mice. These results suggest that abnormal *Rik* expression may influence *Bmp4* signaling related to UB branching to reduce the number of UB branches in mice, thus leading to the development of CAKUT. Additional studies are needed to identify other molecules regulated by *Rik*.

Variability in the cellular localization of lncRNAs can regulate gene expression by various mechanisms. For example, cytoplasmic lncRNAs can act as a miRNA sponge, competitively inhibit miRNA, reduce degradation of mRNA by miRNA, and regulate mRNA stability and translation (Beermann et al., 2016; Yao et al., 2019). Nuclear lncRNAs mainly regulate the expression of their target genes at the transcriptional level and can bind to the promoter regions of the target genes or interact with transcription factors, thereby inhibiting or promoting expression of the adjacent target genes (Latos et al., 2012; Engreitz et al., 2016; Cho et al., 2018; Wang et al., 2019). The subcellular localization experiments performed in the present study suggest that *Rik* is located in the nucleus. We speculate that *Rik* may play a biological role by influencing the expression of the adjacent genes and found that *Bmp4* expression is downregulated by *Rik* overexpression. Therefore, we suggest that *Rik*, which is located adjacent to *Bmp4* in the genome, may inhibit the expression of *Bmp4* via a certain transcription factor or by regulating the promoter of the *Bmp4* gene. Specific mechanisms of the regulation of *Bmp4* by *Rik* require additional investigation.

## Conclusion

In summary, this study found that overexpression of the lncRNA *Rik* induced the development of CAKUT in mice, predominantly involving abnormal ureteral bud branching; abnormal urinary development may be mediated by *Rik*-dependent regulation of the expression of *Bmp4*, a key molecule in the UB branching. This study provides new evidence of the involvement of lncRNAs in the process of embryonic development and new insight into investigations of the etiological mechanisms of CAKUT.

## Data Availability Statement

The raw data supporting the conclusions of this article will be made available by the authors, without undue reservation.

## Ethics Statement

The animal study was reviewed and approved by the animal welfare and usage management regulations of the School of Life Sciences of Fudan University [Protocol Approval No. SYXK (hu) 2020-0011].

## Author Contributions

LT, HX, and QS designed this study. LT completed the experiment, analyzed the data, and wrote the first draft. JC, YZ, XF, and XW interpreted the experimental results. JL, JJL, YL, and SX analyzed the images. MY and QS revised and refined the manuscript. QS edited and approved the final version. All authors contributed to the article and approved the submitted version.

## Conflict of Interest

The authors declare that the research was conducted in the absence of any commercial or financial relationships that could be construed as a potential conflict of interest.

## Footnotes

1 http://www.geneontology.org/

2 https://en.wikipedia.org/wiki/Hypergeometric_distribution
