# Supplementary figures and images for: Overexpression of Long Non-coding RNA 4933425B07Rik Causes Urinary Malformations in Mice
Source: Front Cell Dev Biol. 2021 Feb 19;9:594640. doi: 10.3389/fcell.2021.594640 (PMC7933199; doi:10.3389/fcell.2021.594640)

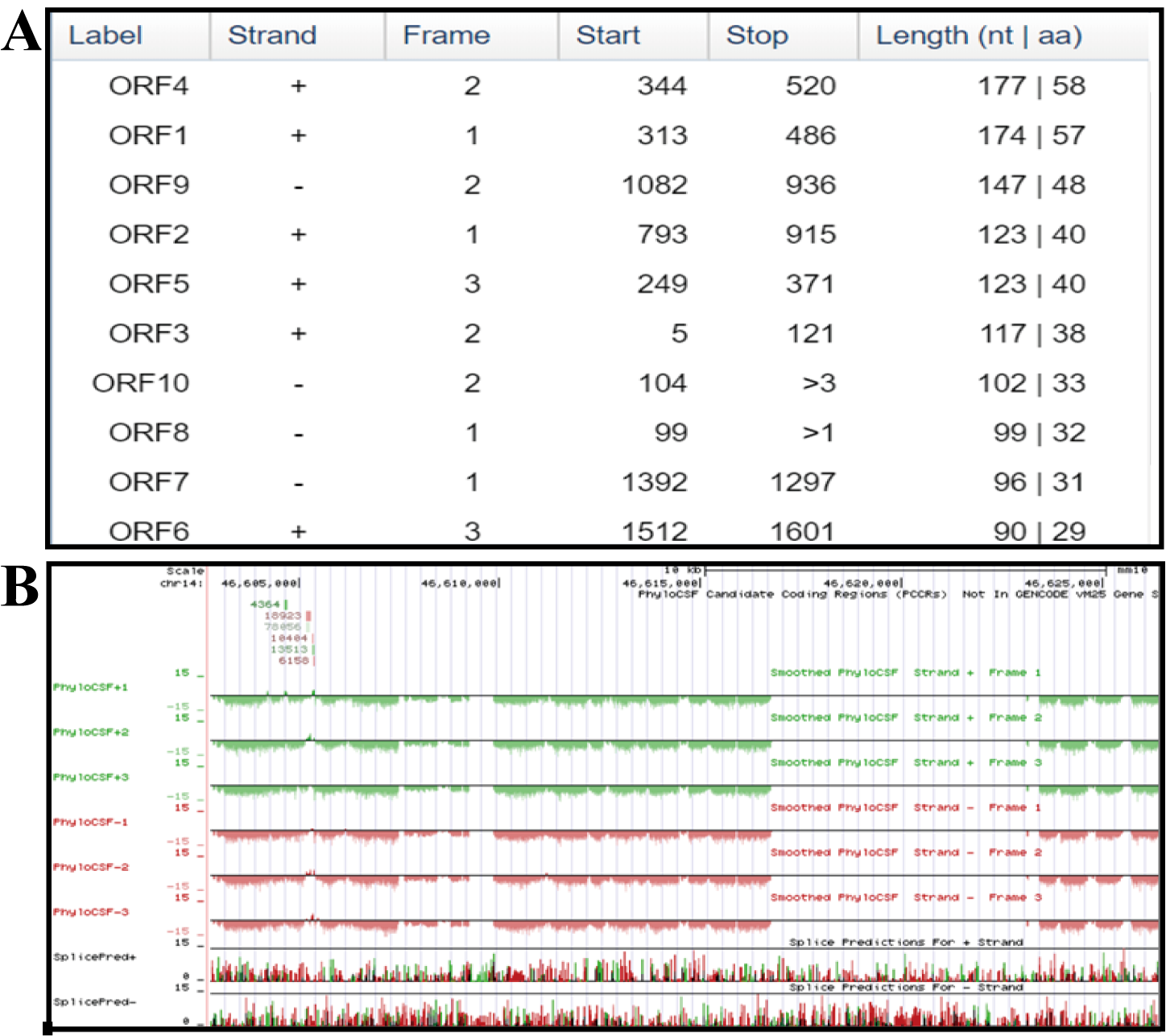

Supplement: Supplementary file 1 [file Image_1.TIF]

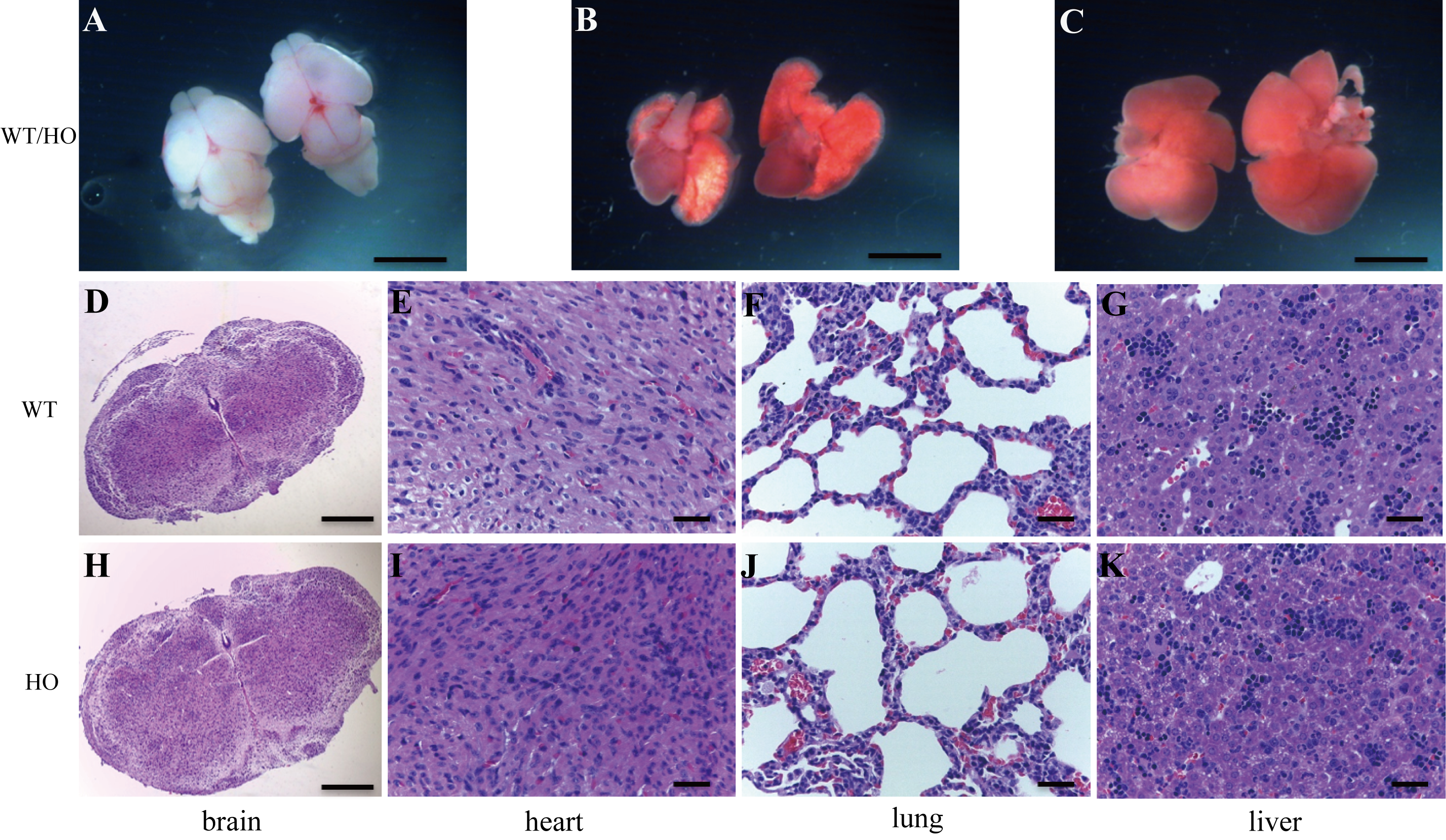

Supplement: Supplementary file 2 [file Image_2.TIF]
