# Supplementary material for: Overexpression of Long Non-coding RNA 4933425B07Rik Causes Urinary Malformations in Mice
Source: Front Cell Dev Biol. 2021 Feb 19;9:594640. doi: 10.3389/fcell.2021.594640 (PMC7933199; doi:10.3389/fcell.2021.594640)
Supplement: Supplementary file 3 [file Table_1.docx]

Supplementary Material

**Supplemental Figure S1.** *Rik* protein-coding potential prediction. (A) Prediction of putative proteins encoded by lncRNA *Rik* using ORF Finder. ORF, open reading frame. (B) Coding potency of lncRNA *Rik* using PhyloCSF. Scores above 0 suggest coding potential, whereas scores below 0 represent no coding potential. The results display no protein-coding potential in lncRNA *Rik*.

**Supplemental Figure S2.** No significant abnormalities were observed in the general anatomy and histopathology of the brain, heart, lung, and liver of *Rik*^PB/PB^ and *Rik*^+/+^ newborn mice. (A-C) Gross anatomical morphology of *Rik*^+/+^ mice (left) and *Rik*^PB/PB^ mice (right). (D-K) Histopathological structure; (D, H) brain, (E, I) heart, (F, J) lung, (G, K) liver. Scar bars, 50mm in A-C; 5mm in D and H; 500um in E-G and I-K.

**Supplemental Table 1.** RNA-seq results of important markers for UB branching at E12.5 wild type and *Rik*^PB/PB^ kidneys.

| \| Gene \| WT \| HO \| Fold change \| P value \| Significance \| \| --- \| --- \| --- \| --- \| --- \| --- \| \| *4933425B07Rik* \| 1.01733 \| 132.21374 \| 129.96151 \| 0.001 \| yes \| \| *Foxc1* \| 29.76333 \| 35.64333 \| 1.19756 \| 0.007 \| yes \| \| *Foxc2* \| 52.93333 \| 62.23333 \| 1.17569 \| 0.001 \| yes \| \| *Wnt9b* \| 3.78333 \| 4.70667 \| 1.24405 \| 0.003 \| yes \| \| *Gata3* \| 10.95667 \| 12.96333 \| 1.18315 \| 0.009 \| yes \| \| *Bmp4* \| 14.51667 \| 6.94 \| 0.47807 \| 0.001 \| yes \| \| *Islet (Isl1)* \| 0.86 \| 1.15333 \| 1.33721 \| 0.649 \| no \| \| *Six2* \| 118.89667 \| 120.77667 \| 1.01581 \| 0.815 \| no \| \| *Gdnf* \| 18 \| 17.61 \| 0.97833 \| 0.786 \| no \| \| *Bmp7* \| 29.86667 \| 33.60333 \| 1.12511 \| 0.102 \| no \| \| *Gfrα1* \| 29.01333 \| 30.13333 \| 1.03860 \| 0.633 \| no \| \| *Wnt4* \| 13.85333 \| 11.77 \| 0.84962 \| 0.250 \| no \| \| *Ret* \| 9.83667 \| 9.04667 \| 0.91969 \| 0.438 \| no \| \| *Agtr2* \| 19.38667 \| 20.53667 \| 1.05932 \| 0.847 \| no \| \| *Etv5* \| 30.19333 \| 28.53667 \| 0.94513 \| 0.581 \| no \| \| *Etv4* \| 51.68 \| 44.91 \| 0.86900 \| 0.135 \| no \| \| *Gremlin1* \| 0.32 \| 0.33333 \| 1.04166 \| 0.975 \| no \| \| *Wnt11* \| 15.92333 \| 14.16 \| 0.88926 \| 0.177 \| no \| \| *Sall1* \| 79.20333 \| 74.86333 \| 0.94520 \| 0.449 \| no \| \| Agtr1b \| 0.04667 \| 0.06333 \| 1.35697 \| 0.519 \| no \| \| *Agt* \| 0.02 \| 0.02667 \| 1.3335 \| 0.069 \| no \| \| *Wnt2b* \| 1.06 \| 1.57 \| 1.48113 \| 0.199 \| no \| \| *Pbx1* \| 118.61 \| 132.2 \| 1.11458 \| 0.062 \| no \| \| *Fgf7* \| 1.12 \| 0.73 \| 0.65179 \| 0.091 \| no \| \| *Six1* \| 0.91667 \| 1.37667 \| 1.50182 \| 0.279 \| no \| |
| --- | --- | --- | --- | --- | --- | --- | --- | --- | --- | --- | --- | --- | --- | --- | --- | --- | --- | --- | --- | --- | --- | --- | --- | --- | --- | --- | --- | --- | --- | --- | --- | --- | --- | --- | --- | --- | --- | --- | --- | --- | --- | --- | --- | --- | --- | --- | --- | --- | --- | --- | --- | --- | --- | --- | --- | --- | --- | --- | --- | --- | --- | --- | --- | --- | --- | --- | --- | --- | --- | --- | --- | --- | --- | --- | --- | --- | --- | --- | --- | --- | --- | --- | --- | --- | --- | --- | --- | --- | --- | --- | --- | --- | --- | --- | --- | --- | --- | --- | --- | --- | --- | --- | --- | --- | --- | --- | --- | --- | --- | --- | --- | --- | --- | --- | --- | --- | --- | --- | --- | --- | --- | --- | --- | --- | --- | --- | --- | --- | --- | --- | --- | --- | --- | --- | --- | --- | --- | --- | --- | --- | --- | --- | --- | --- | --- | --- | --- | --- | --- | --- | --- | --- | --- | --- | --- | --- |

WT, wild type; HO, homozygous.
